# Estimated glucose disposal rate and non-HDL-c/HDL-c ratio with the progression of carotid atherosclerosis: a long-term cohort study

**DOI:** 10.3389/fmed.2025.1627246

**Published:** 2025-07-17

**Authors:** Bingqing Han, Jing Ma, Shanshan Liu, Chao Fu, Hao Zhang, Yi Luo, Fei Wang, Qiang Zeng

**Affiliations:** ^1^School of Medicine, Nankai University, Tianjin, China; ^2^Health Management Institute, The Second Medical Center & National Clinical Research Center for Geriatric Diseases, Chinese PLA General Hospital, Beijing, China

**Keywords:** estimated glucose disposal rate, Non-HDL-c/HDL-c ratio, insulin resistance, carotid atherosclerosis, metabolism

## Abstract

**Background:**

Both the estimated glucose disposal rate (eGDR) and the non-HDL-c/HDL-c ratio (NHHR) are associated with cardiovascular disease risk and prognosis. It is unclear whether assessing eGDR and NHHR together improves CAS progression prediction.

**Methods:**

This large cross-sectional and longitudinal cohort study included 7,360 adults who underwent multiple health check-ups at the Chinese PLA General Hospital from October 2009 to December 2023. The relationships of the eGDR and NHHR with CAS progression were determined through multivariable Cox regression analysis and restricted cubic splines (RCS).

**Results:**

During a median follow-up period of 30 months, we included 7,360 participants. The restricted cubic spline curve of the correlation between the eGDR and CAS progression was non-linear. There was a positive linear relationship between the NHHR and CAS progression. When the eGDR was <8.71 (median level) mg/kg/min and the NHHR was >2.89, the risk of CAS progression significantly increased. Subgroup analysis revealed that age significantly altered the correlation. The incorporation of the eGDR and NHHR into the basic model significantly enhanced the usefulness of the model for predicting CAS progression. Furthermore, mediation analysis revealed that the NHHR significantly mediated the impact of the eGDR on CAS progression.

**Conclusions:**

This study revealed that a lower eGDR and higher NHHR are associated with an increased risk of CAS progression. The combined assessment of the eGDR and NHHR can enhance the identification of high-risk populations, which is useful for the implementation of active preventive measures.

## Background

Atherosclerotic cardiovascular disease (ASCVD), especially coronary heart disease, is the leading cause of death in developed countries and some developing countries, resulting in a significant economic and social burden (1). Research indicates that in the secondary prevention population, even if modifiable risk factors meet the guideline-recommended targets, some patients will still have a recurrence risk of over 20% or even over 30% (2). Therefore, improving non-traditional risk factors is crucial for further reducing the burden of ASCVD. Insulin resistance (IR) plays a very important role in the development of CVDs and is an independent risk factor for CVDs and adverse CV outcomes (3–5). In patients with type 1 diabetes, IR markers, especially eGDR, are significantly associated with carotid atherosclerosis (6). The current “gold standard” for assessing IR is the hyperinsulinemic–euglycemic clamp technique (HEC), but its operation is complex, expensive, and invasive, making it unsuitable for population screening. The triglyceride–glucose index (TyG index) is also considered a reliable indicator of IR (7, 8). The TYG index has been widely used in many studies in recent years to predict the prognosis of CVDs (9–11). Moreover, the acute phase of some ASCVD diseases may lead to stress-induced hyperglycemia, and dietary changes can also cause variations in triglycerides (TGs) or fasting blood glucose (FBG), which may affect the diagnostic or predictive value of the TyG index based on the TyG formula. Moreover, the TYG index does not account for other indicators closely related to IR, such as central obesity and hypertension (HTN) (12). The estimated glucose disposal rate (eGDR), which is based on waist circumference (WC), HTN, and glycosylated hemoglobin (HbA1c), can be used not only to assess IR in individuals with type 1 diabetes (T1DM) but also to predict the occurrence and prognosis of adverse CV and cerebrovascular events in patients with type 2 diabetes (T2DM) and non-diabetic individuals (13, 14). Abnormal glucose metabolism is often accompanied by abnormal lipid metabolism (15). In vitro and in vivo studies have shown that high concentrations of insulin can stimulate de novo lipogenesis, leading to increased synthesis and secretion of very low-density lipoproteins by activating SREBP-1C and inhibiting acetyl-CoA carboxylase (16). Some non-traditional lipid parameters can provide more information than conventional parameters and can better reflect the interactions between lipid components (17). The non-HDL-c/HDL-c ratio (NHHR) includes information on promoting atherosclerosis and preventing atherosclerosis. A study of the elderly population in China suggested that maintaining an NHHR below 2.685 may significantly reduce the risk of stroke (18). The calculation method of eGDR does not include lipid indicators, which may overlook the impact of lipids on carotid atherosclerosis. The combined assessment of NHHR and eGDR can address this shortcoming.

Therefore, considering that IR and dyslipidemia are two important indicators of metabolic syndrome, we conducted a longitudinal study based on data from a retrospective cohort of a general health examination population. The aim of this study was to explore the joint effects and risk reclassification ability of the eGDR and NHHR on the progression of carotid atherosclerosis (CAS). Furthermore, we emphasized the dual mediating effect of the eGDR and NHHR on the progression of CAS.

## Methods

### Study population

**Figure 1:**
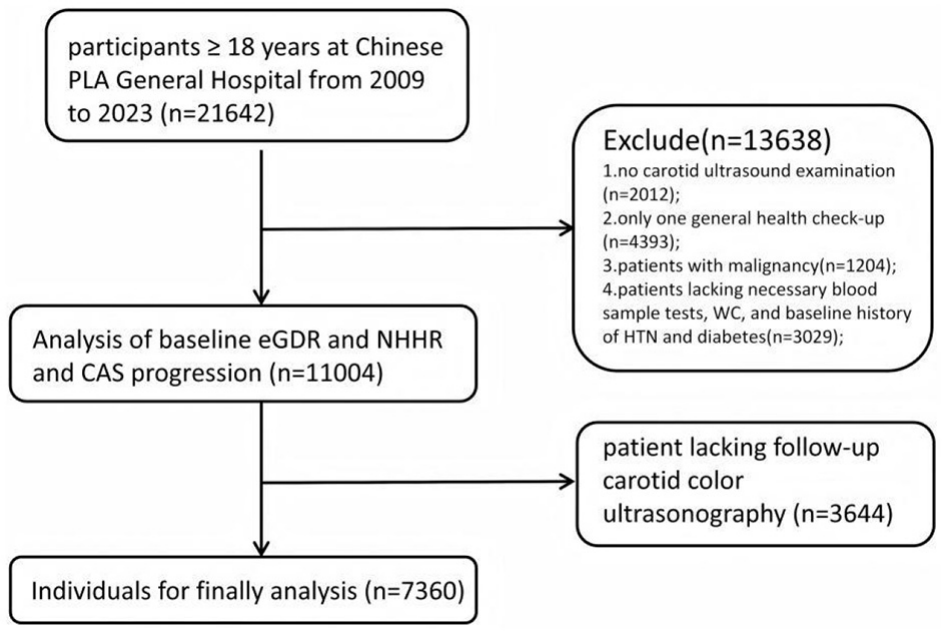
Flowchart of study participants. CAS, carotid atherosclerosis; eGDR, estimated glucose disposal rate; NHHR, non-HDL-C/HDL-C ratio.

This retrospective, population-based longitudinal cohort study is based on a general health check-up population from the Department of Health Medicine at the Chinese PLA General Hospital. From October 2009 to December 2023, a total of 21,642 participants aged 18 and above underwent general health check-ups. The exclusion criteria were as follows: (1) no carotid ultrasound examination (*n* = 2,012); (2) only one general health check-up (*n* = 4,393); (3) patients lacking necessary blood sample tests, WC, and baseline history of HTN and diabetes (*n* = 3,029); (4) patients with malignancy (*n* = 1,204); and (5) patient lacking follow-up carotid color ultrasonography (*n* = 3,644). In the end, a total of 7,360 participants were included in the final analysis and further divided into four subgroups on the basis of the quartiles (Qs) of the eGDR. The detailed inclusion and exclusion process is shown in Figure 1. This study was conducted in accordance with the Declaration of Helsinki and approved by the Ethics Committee of the Chinese PLA General Hospital (S2019-190-02). All patients provided written informed consent.

### Characteristics and definition

Clinical data were collected from the enrolled patients, and their general information and medical history were recorded. Researchers also measured participants' weight, height, and WC while they were wearing light clothing and not wearing shoes. The body mass index (BMI) was calculated as weight (kg)/height (m)^2^, and obesity was defined as a BMI ≥ 28 kg/m^2^. The definition of HTN is a systolic blood pressure ≥140 mmHg or a diastolic blood pressure ≥90 mmHg, current use of antihypertensive medication, or self-reported history of HTN. The definition of diabetes was FBG ≥7.0 mmol/L in the cohort examination or a self-reported history of diabetes diagnosed by a doctor (19). In addition, the biochemical parameters tested included HbA1c, total cholesterol (TC), TGs, low-density lipoprotein cholesterol (LDL-C), high-density lipoprotein cholesterol (HDL-C), FBG, high-sensitivity C-reactive protein (hsCRP), and blood creatinine. The eGDR (mg/kg/min) was calculated with the following formula: 21.158–(0.09^*^WC)–(3.407^*^HTN)–(0.551^*^HbA1c) (20). The NHHR data is obtained using the formula for TC minus HDL-C, then divided by HDL-C. Fasting blood samples were collected in the morning via a Roche C8000 fully automated biochemical analyzer (Roche, Mannheim, Germany) equipped with corresponding reagents, calibrators, and quality control materials to analyze a series of biochemical parameters.

### Carotid ultrasonography and study outcomes

The ultrasound evaluation of the bilateral carotid arteries was manually performed by certified physicians from the Department of Health Medicine at the Second Medical Center of the PLA General Hospital, who were unaware of this study. The participants were examined via a high-frequency ultrasound probe (7.5–10.0 MHz). Abnormal carotid intima–media thickness (cIMT) is defined as a maximum cIMT value ≥0.9 mm, which is the maximum distance between the intima-media and the outer membrane of the lumen. Carotid plaques were defined as cIMT ≥ 1.5 mm, focal structures protruding into the arterial lumen ≥0.5 mm, or ≥50% of the surrounding cIMT value. Furthermore, the progression of CAS is defined as the emergence of new carotid artery stenosis, carotid artery plaques, or cIMT during the follow-up period compared with baseline. For individuals with combined carotid plaques and cIMT, baseline data and follow-up results are defined on the basis of the dominant manifestation (i.e., carotid plaques) (21).

### Statistical analysis

Continuous variables were analyzed using ANOVA for normally distributed data and the Kruskal-Wallis H test for skewed distributions, with results expressed as mean ± standard deviation (SD) or median (minimum-maximum range) respectively. Categorical data are reported as frequency counts (percentages), and between-group comparisons were analyzed by chi-square (χ^2^) tests. Proportional hazards assumptions were confirmed using Schoenfeld residuals prior to Kaplan-Meier analysis. Survival probabilities across eGDR quartile groups were estimated using Kaplan-Meier analysis, with between-group statistical differences evaluated by log-rank tests. The adjusted covariates comprised demographic factors (age, sex), anthropometric measures (body mass index), lifestyle variables (smoking, alcohol consumption), clinical comorbidities (hypertension, diabetes), and biochemical parameters (triglycerides, hemoglobin, uric acid, high-sensitivity C-reactive protein). Covariate selection was based on clinical relevance and prior evidence from observational cohort studies.

The dose-response relationships of both eGDR and NHHR with CAS progression were modeled using restricted cubic splines (RCS) with five knots. The optimal knot positions in the restricted cubic spline (RCS) models were determined through Akaike Information Criterion (AIC) minimization. Kaplan-Meier and Cox regression models were used to analyze the associations of eGDR and NHHR with the progression of CAS.

Mediation analysis was used to investigate the potential mediating role of NHHR in the association between eGDR and CAS progression. Stratified analyses assessed effect heterogeneity across clinically relevant subgroups: age (< 60 vs. ≥60 years), sex, smoking status, and alcohol use, with multiplicative interaction terms evaluated through likelihood ratio tests. We also performed interaction analyses to assess the potential interactions between each subgroup and CAS progression.

We evaluated the robustness of our conclusions by conducting several sensitivity analyses. First, the analysis was repeated after excluding subjects with diabetes. Second, hypertension was redefined using a threshold of 130/80 mmHg, and eGDR was recalculated to reanalyze the data for the remaining subjects. The incremental predictive capacity of eGDR and NHHR for CAS progression was assessed through Harrell's C-statistic, with model discrimination improvements quantified using continuous net reclassification improvement (NRI) and integrated discrimination improvement (IDI) indices. All statistical analyses were performed using R software version 4.4.2. All inferential analyses employed two-tailed hypothesis testing with α = 0.05 as the significance threshold.

## Results

### Baseline characteristics according to quartiles of eGDR

Among the 7,360 eligible participants, the average age was 49.3 ± 8.72 years, with 31.0% being female. The median eGDR was 8.71, and the median follow-up time was 30 months. A total of 3,751 patients (51.0%) experienced outcome events. The baseline characteristics of the individuals included were based on CAS progression (see Supplementary Table S1). A comparison of the baseline characteristics stratified by eGDR quartiles (Q1: 0.330–6.139; Q2: 6.141–8.711; Q3: 8.712–10.498; and Q4: 10.507–13.403) is shown in Table 1. The average age, male sex ratio, systolic blood pressure, diastolic blood pressure, BMI, WC, HGB, HbA1c, TG, LDL, UA, and hsCRP levels, current smoking status, and current alcohol consumption status all decreased with an increasing eGDR (all *P* < 0.001).

**Table 1:** Baseline characteristics of study participants according to quartiles of eGDR.

**Characteristics**	**Overall**	**Quartiles of eGDR**				***P* value**
		**Q1 (0.33–6.14)**	**Q2 (6.14–8.71)**	**Q3 (8.71–10.40)**	**Q4 (10.51–13.40)**	
*N*	7,360	1,842	1,839	1,841	1,838	
Female, *n* (%)	2,283 (31.02%)	190 (10.31%)	444 (24.14%)	306 (16.62%)	1,343 (73.07%)	< 0.001
Age, years	49.3 ± 8.72	52.0 ± 8.48	51.2 ± 8.49	48.8 ± 7.82	45.3 ± 8.47	< 0.001
Diabetes, *n* (%)	1,470 (19.97%)	709 (38.49%)	391 (21.26%)	298 (16.19%)	72 (3.92%)	< 0.001
SBP, mmHg	121 ± 18.40	135 ± 16.30	128 ± 16.40	116 ± 12.90	106 ± 13.40	< 0.001
DBP, mmHg	79.3 ± 11.70	87.0 ± 11.50	83.0 ± 10.90	75.4 ± 8.24	71.6 ± 8.74	< 0.001
Current smoking, *n* (%)	2,741 (37.24%)	902 (48.97%)	740 (40.24%)	846 (45.95%)	253 (13.76%)	< 0.001
Current drinking, *n* (%)	4,501 (61.15%)	1,464 (79.47%)	1,210 (65.80%)	1,266 (68.77%)	561 (30.52%)	< 0.001
BMI, kg/m^2^	25.6 ± 3.65	28.6 ± 2.99	25.9 ± 2.99	26.0 ± 2.53	21.8 ± 2.27	< 0.001
WC, cm	89.5 ± 11.90	100 ± 7.09	89.9 ± 9.57	93.0 ± 6.14	74.8 ± 6.24	< 0.001
NHHR	2.89 (0.40–7.96)	3.27 (0.44–7.96)	3.00 (0.51–7.54)	3.22 (0.40–7.82)	2.16 (0.49–6.61)	< 0.001
FBG, mmol/L	5.73 ± 1.42	6.46 ± 1.80	5.82 ± 1.53	5.62 ± 1.10	5.00 ± 0.47	< 0.001
HbA1c, %	5.82 ± 0.86	6.28 ± 1.06	5.87 ± 0.98	5.76 ± 0.61	5.37 ± 0.32	< 0.001
Hemoglobin, g/L	145 ± 15.50	151 ± 12.40	147 ± 14.50	149 ± 13.30	134 ± 16.00	< 0.001
TC, mmol/L	4.69± 0.93	4.64 ± 0.99	4.70 ± 0.96	4.77 ± 0.87	4.66 ± 0.87	< 0.001
TG, mmol/L	1.45 (0.28–15.50)	1.83 (0.30–15.50)	1.55 (0.32–10.70)	1.55 (0.36–12.90)	0.98 (0.28–7.48)	< 0.001
LDL-C, mmol/L	3.10 ± 0.85	3.05 ± 0.91	3.10 ± 0.88	3.21 ± 0.80	3.02 ± 0.79	< 0.001
HDL-C, mmol/L	1.24 ± 0.35	1.11 ± 0.26	1.20 ± 0.33	1.16 ± 0.30	1.48 ± 0.39	< 0.001
UA, μmol/L	347 ± 89.60	381 ± 83.40	357 ± 85.40	367 ± 81.80	283 ± 73.30	< 0.001
hsCRP, mg/L	0.11 (0–17.70)	0.14 (0–16.50)	0.11 (0–17.70)	0.11 (0–4.92)	0.08 (0–2.82)	< 0.001
Lipid-lowering medications, *n* (%)	235 (12.8%)	155 (8.4%)	81 (4.4%)	18 (1.0%)	489 (6.6%)	< 0.001
Antidiabetic medications, *n* (%)	406 (22.0%)	221 (12.0%)	154 (8.4%)	8 (0.4%)	789 (10.7%)	< 0.001

BMI, body mass index; DBP, diastolic blood pressure; SBP, systolic blood pressure; eGDR, estimated glucose disposal rate; NHHR, non-HDL-C/HDL-C ratio; FBG, fasting blood glucose; HbA1c, glycosylated hemoglobin A1c; HDL-C, high density lipoprotein cholesterol; hsCRP, high-sensitivity C-reactive protein; LDL-C, low density lipoprotein cholesterol; TC, total cholesterol; TG, triglycerides; UA, uric acid; WC, waist circumference.

### eGDR, NHHR, and CAS progression

**Figure 2:**
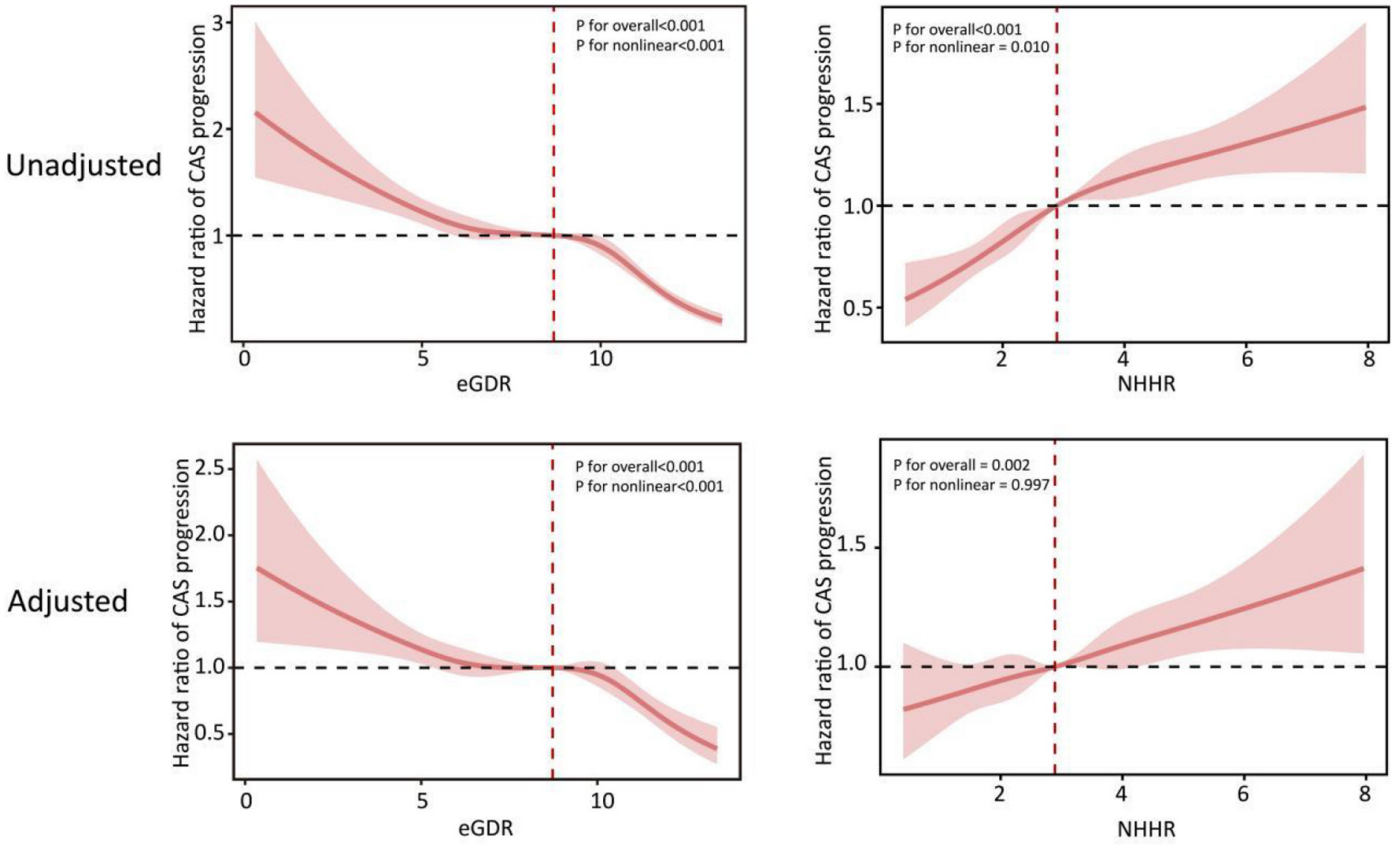
Restricted cubic spline curves for CAS progression according to the eGDR and NHHR. Hazard ratios are indicated by solid lines and 95% CIs by shaded areas. The horizontal dotted line represents the hazard ratio of 1.0. The adjusted models age, sex, BMI, current smoking, current drinking, TG, HGB, UA, and hs-CRP. eGDR, estimated glucose disposal rate; NHHR, non-HDL-C/HDL-C ratio.

**Figure 3:**
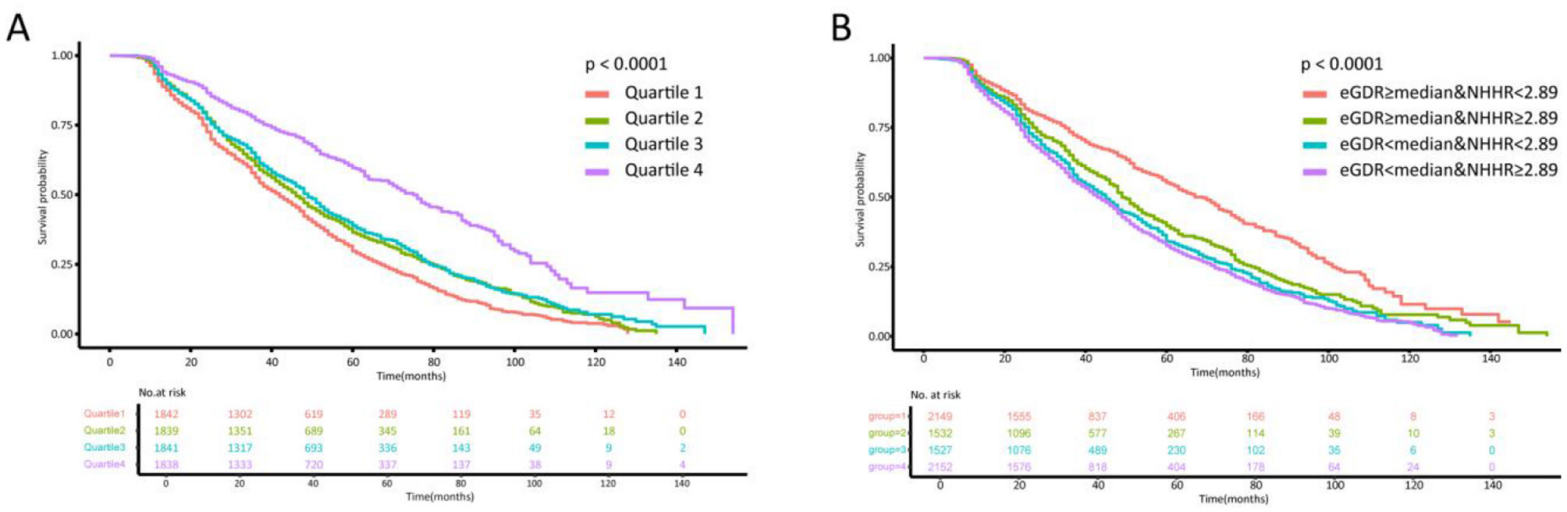
**(A)** Kaplan–Meier survival curves for CAS progression with different quartile levels of baseline eGDR. eGDR: Q1 (0.330–6.139), Q2 (6.141–8.711), Q3 (8.712–10.498), and Q4 (10.507-−13.403). **(B)** Kaplan–Meier survival curves for CAS progression by eGDR and NHHR level. eGDR, estimated glucose disposal rate; NHHR, non-HDL-C/HDL-C ratio; Group 1: eGDR ≥ median & NHHR <2.89; Group 2: eGDR ≥ median & NHHR ≥2.89; Group 3: eGDR < median & NHHR <2.89; Group 4: eGDR < median & NHHR ≥2.89. median of eGDR: 8.711.

According to restricted cubic spline analysis, the eGDR in the population showed a non-linear relationship with CAS progression, whereas the NHHR showed a linear positive correlation with CAS progression. After fully adjusting for covariates, the eGDR still exhibited a non-linear relationship with CAS progression (overall *P* < 0.001, non-linear *P* < 0.001). After redefining HTN as ≥130/80 mmHg, the eGDR showed a linear negative correlation with CAS progression (Supplementary Figure S1). The restricted cubic spline plot shows that the cutoff value of NHHR is 2.89. When the NHHR were >2.89, the risk of CAS progression significantly increased (Figure 2). Some previous studies have also shown that the high-risk threshold of NHHR is around 2.8. Therefore, we selected 2.89 as the high-risk threshold for NHHR for subsequent analysis (22, 23). The restricted cubic spline plot shows that the cutoff value of eGDR is 8.73. When the eGDR were < 8.73, the risk of CAS progression significantly increased (Figure 2). However, since eGDR was nonlinearly correlated with the progression of carotid atherosclerosis, we finally selected a median of 8.71 as the high-risk threshold for subsequent analysis. As shown in Table 2 and Figure 3A, the Kaplan–Meier survival curve indicated that individuals with a lower eGDR had a greater risk of CAS progression. Compared with those with an eGDR in Q1, the HRs (95% CI) for CAS progression with an eGDR in Q2-4 were 0.82 (0.75–0.89), 0.78 (0.71–0.85), and 0.44 (0.40–0.49), respectively. After adjusting for factors such as age, sex, smoking status, alcohol consumption status, and UA levels, the results were similar. When jointly assessing the progression of CAS at the baseline eGDR index and NHHR values, the lower the population eGDR index and the higher the NHHR value were, the greater the risk of CAS progression was (Figure 3B, Table 3).

**Table 2:** Hazard ratios (95% confidence intervals) of CAS progression by baseline eGDR.

**eGDR**	**Unadjusted**		**Model 1**		**Model 2**	
	**HR (95% CI)**	***P*** **value**	**HR (95% CI)**	***P*** **value**	**HR (95% CI)**	***P*** **value**
Quartile 1	Reference		Reference		Reference	
Quartile 2	0.82 (0.75–0.89)	< 0.001	0.86 (0.79–0.93)	< 0.001	0.88 (0.81–0.97)	0.007
Quartile 3	0.78 (0.71–0.85)	< 0.001	0.85 (0.78–0.93)	< 0.001	0.88 (0.80–0.97)	0.007
Quartile 4	0.44 (0.40–0.49)	< 0.001	0.61 (0.54–0.69)	< 0.001	0.67 (0.58–0.77)	< 0.001

Model 1: Adjusted for age and sex; Model 2: Adjusted for age, sex, BMI, current smoking, current drinking, TG, HGB, UA, hs-CRP. CAS, carotid atherosclerosis progression; eGDR, estimated glucose disposal rate; HGB, Hemoglobin; hsCRP, high-sensitivity C-reactive protein; HR, hazard ratio; UA, uric acid; CI, confidence interval.

**Table 3:** Risk of CAS progression upon individual exposure stratified by eGDR and NHHR.

	**Unadjusted**		**Model 1**		**Model 2**	
**Groups**	**HR (95% CI)**	***P*** **value**	**HR (95% CI)**	***P*** **value**	**HR (95% CI)**	***P*** **value**
eGDR ≥ median	Reference		Reference		Reference	
eGDR < median	1.48 (1.39–1.58)	< 0.001	1.20 (1.12–1.29)	< 0.001	1.12 (1.04–1.20)	0.004
NHHR < 2.89	Reference		Ref		Ref	
NHHR ≥ 2.89	1.32 (1.23–1.40)	< 0.001	1.18 (1.10–1.26)	< 0.001	1.09 (1.01–1.17)	0.025
eGDR ≥ median & NHHR < 2.89	Reference		Ref		Ref	
eGDR ≥ median & NHHR ≥ 2.89	1.44 (1.30–1.59)	< 0.001	1.24 (1.12–1.37)	< 0.001	1.15 (1.03–1.28)	0.012
eGDR < median & NHHR < 2.89	1.66 (1.50–1.83)	< 0.001	1.26 (1.14–1.40)	< 0.001	1.17 (1.05–1.31)	0.004
eGDR < median & NHHR ≥ 2.89	1.80 (1.65–1.97)	< 0.001	1.40 (1.27–1.54)	< 0.001	1.23 (1.11–1.38)	< 0.001

Model 1: Adjusted for age and sex; Model 2: Adjusted for age, sex, BMI, current smoking, current drinking, TG, HGB, UA, hs-CRP. CAS, carotid atherosclerosis progression; eGDR, estimated glucose disposal rate; HGB, Hemoglobin; hsCRP, high-sensitivity C-reactive protein; HR, hazard ratio; UA, uric acid; CI, confidence interval; median of eGDR: 8.711.

### Mediation analyses

**Figure 4:**
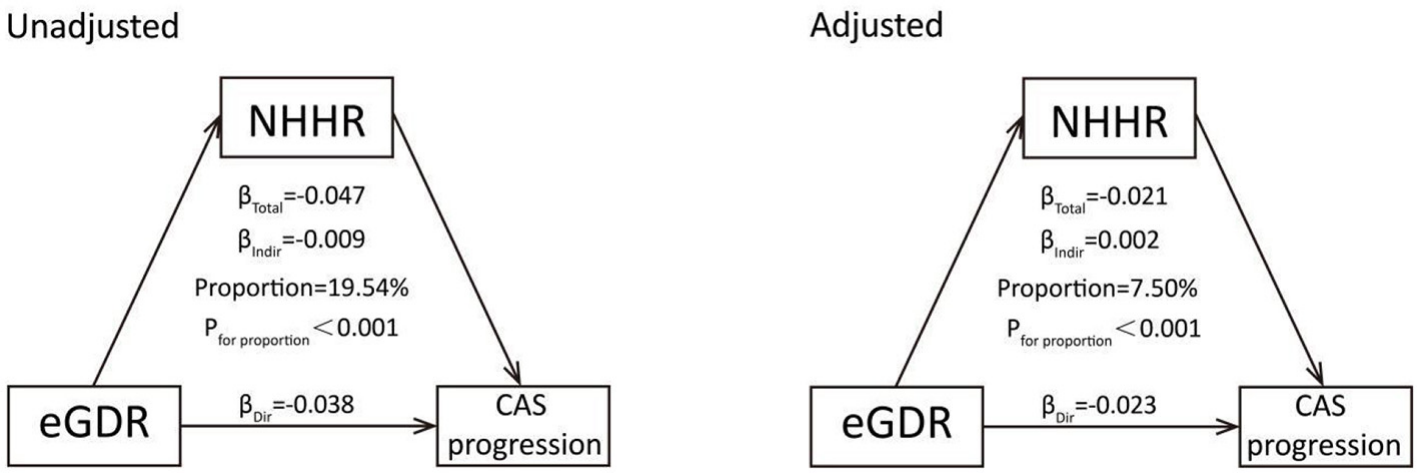
Mutual mediation effects of the eGDR and NHHR on CAS progression. Adjusted for age, sex, BMI, current smoking, current drinking, TG, HGB, UA, and hs-CRP. CAS, carotid atherosclerosis progression; eGDR, estimated glucose disposal rate; HGB, Hemoglobin; hs-CRP, high-sensitivity C-reactive protein; NHHR, non-HDL-C/HDL-C ratio.

We used mediation analysis to further clarify the reciprocal mediating effects of the eGDR and NHHR on the progression of CAS (Figure 4, Supplementary Figure S2). According to the unadjusted model, the NHHR significantly mediated the effect of the eGDR on the risk of CAS progression (proportion = 19.54%). After fully adjusting for covariates, the NHHR still considerably mediated the effect of the eGDR on the risk of CAS progression (proportion = 7.50%). Similarly, the eGDR also plays an important mediating role in the impact of the NHHR on the progression of CAS (Supplementary Figure S2).

### Subgroup analyses

**Figure 5:**
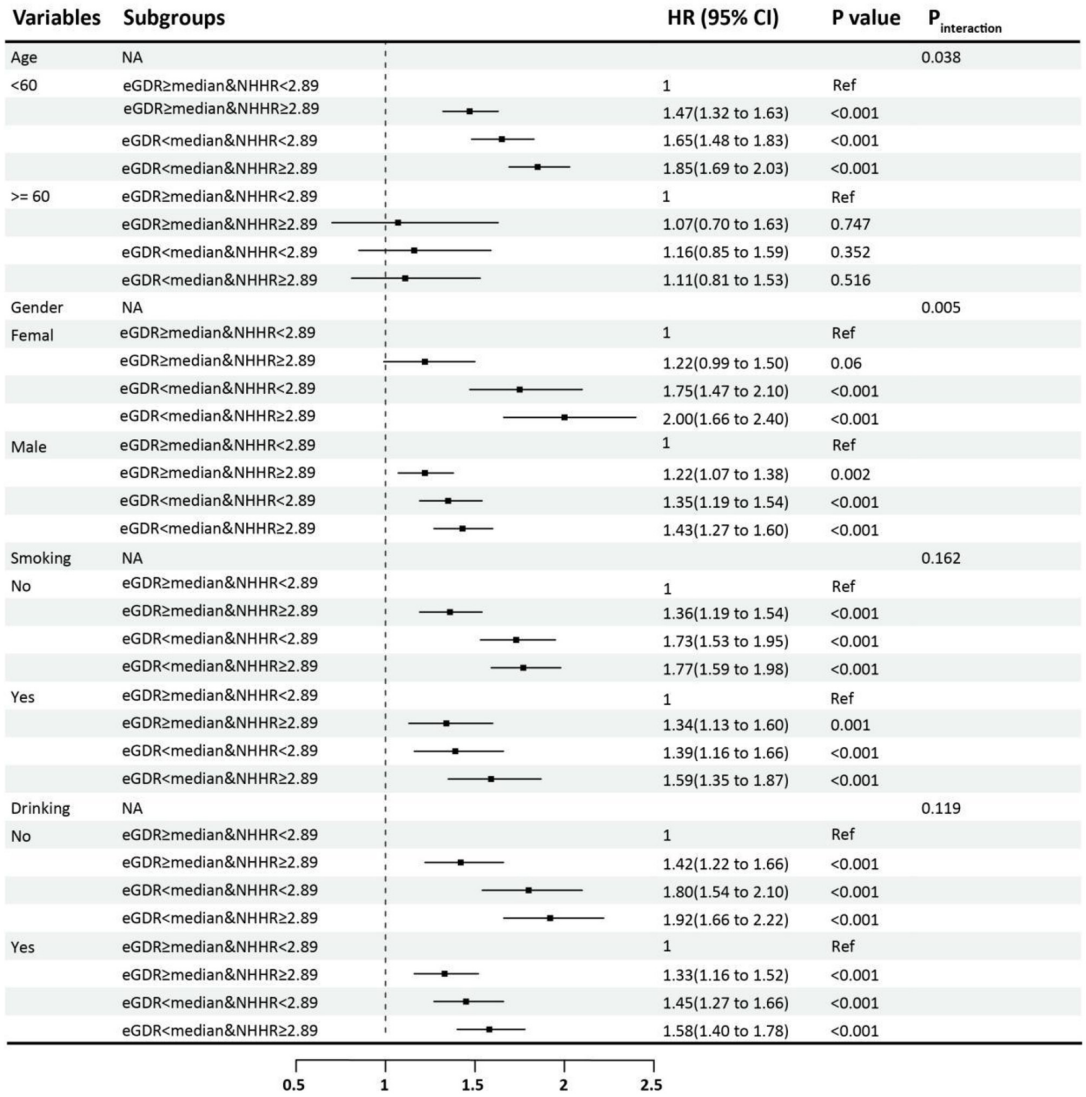
Subgroup and interaction analysis of HRs (95% CIs) for CAS progression of eGDR and NHHR. eGDR, estimated glucose disposal rate; NHHR, non-HDL-C/HDL-C ratio. median of eGDR: 8.711.

The results of the subgroup analysis revealed that in most of the prespecified subgroups, the relationship between eGDR combined with the NHHR and the risk of CAS progression was consistent with the main results (Figure 5). A significant impact on predictive performance was observed in the age subgroup (interaction *P* = 0.038).

### Sensitivity analyses

In the sensitivity analysis, when we excluded diabetes patients defined by FBG and HbA1c measurements, no significant changes were observed in the results (Table 4). When we recalculated the eGDR using the redefined HTN criterion (≥130/80 mmHg), the results remained largely unchanged (Supplementary Table S2). To further reduce the impact of drug treatment on the outcome, we excluded the population using lipid-lowering drugs and hypoglycemic drugs. The result we obtained is consistent with the main result (Supplementary Table S3). Furthermore, to minimize potential selection bias as much as possible, we excluded participants with overly long follow-up periods and those with overly short follow-up periods. The results we obtained are similar to the main results, and these analyses prove the robustness of the main results (Supplementary Table S4).

**Table 4:** The association of eGDR and NHHR with CAS progression among non-diabetic participants (defined diabetic based on FBG, HbA1c and Medical history).

**Groups**	**Unadjusted**		**Model 1**		**Model 2**	
	**HR (95% CI)**	***P*** **value**	**HR (95% CI)**	***P*** **value**	**HR (95% CI)**	***P*** **value**
eGDR ≥ median & NHHR < 2.89	Reference		Reference		Reference	
eGDR ≥ median & NHHR ≥ 2.89	1.44 (1.29–1.60)	< 0.001	1.24 (1.12–1.37)	< 0.001	1.13 (1.00–1.28)	0.042
eGDR < median & NHHR < 2.89	1.66 (1.49–1.86)	< 0.001	1.26 (1.14–1.40)	< 0.001	1.16 (1.03–1.31)	0.017
eGDR < median & NHHR ≥ 2.89	1.82 (1.65–2.01)	< 0.001	1.40 (1.27–1.54)	< 0.001	1.22 (1.08–1.38)	0.002

Model 1: Adjusted for age and sex; Model 2: Adjusted for age, sex, BMI, current smoking, current drinking, TG, HGB, UA, hs-CRP. CAS, carotid atherosclerosis progression; eGDR, estimated glucose disposal rate; NHHR, non-HDL-C/HDL-C ratio. HGB, Hemoglobin; hsCRP, high-sensitivity C-reactive protein; HR, hazard ratio; UA, uric acid; CI, confidence interval; median of eGDR: 8.711.

### Incremental predictive performance of eGDR and NHHR in the CAS progression

Model 3 was used to construct the basic model (including age, sex, BMI, smoking status, alcohol consumption status, and TG). The inclusion of both the eGDR and the NHHR optimized the predictive ability of the basic model for CAS progression (Table 5). Moreover, adding the eGDR and NHHR improved the predictive ability for CAS progression (C statistics: 0.600 vs. 0.597, *P* < 0.001), and all the NRIs in the three models were significant (all *P* < 0.05; Table 5). In addition, we conducted the receiver operating characteristic curves analysis (ROC) to build the basic model (including age, sex, current smoking, current drinking, TC, HDL, TG, LDL, UA, hsCRP). The results show that the area under the curve (AUC) of the basic model is 0.648, the AUC including NHHR is 0.656, the AUC including eGDR is 0.663, and the AUC after including both eGDR and NHHR in the model is 0.670. eGDR+NHHR showed the highest predictive value among the three models and had statistically better discriminative performance (Supplementary Figure S3).

**Table 5:** Improvement in discrimination and risk reclassification for CAS progression after adding eGDR and NHHR.

**Model**	**C-statistic (95% CI)**	***P* value**	**NRI (95% CI)**	***P* value**	**IDI (95% CI)**	***P* value**
Basic model	0.597 (0.587–0.607)	< 0.001	Reference	Reference	Reference	Reference
Basic model+ NHHR	0.599 (0.589–0.609)	< 0.001	0.034 (0.000–0.070)	0.04	0.001 (0.000–0.003)	0.279
Basic model+ eGDR	0.598 (0.589–0.608)	< 0.001	0.072 (0.037–0.109)	< 0.001	0.002 (0.001–0.006)	< 0.001
Basic model+ eGDR +NHHR	0.600 (0.590–0.601)	< 0.001	0.046 (0.011–0.093)	0.01	0.003 (0.001–0.008)	0.01

The basic model included age, sex, BMI, current smoking, current drinking, and TG. CI, confidence interval; IDI, integrated discrimination improvement; NRI, net reclassification improvement; eGDR, estimated glucose disposal rate; NHHR, non-HDL-C/HDL-C ratio.

## Discussion

In this study involving 7,360 adults with a follow-up period of 154 months, we first examined the predictive value of the baseline eGDR and NHHR for the progression of CAS. The main findings are as follows: (1) The lower the eGDR, the higher the NHHR, and the greater the risk of carotid atherosclerosis progression, These correlations are independent of age, gender, smoking, and drinking status; (2) the NHHR played a significant mediating role in the effect of the eGDR on CAS progression and vice versa; and (3) the eGDR and NHHR enhanced the usefulness of the basic model for predicting CAS progression.

IR, a key pathophysiological component of T2DM, is associated with various metabolic disorders, including hyperglycemia, dyslipidemia, and HTN (3). However, patients' glucose and lipid metabolism disorders may interfere with the predictive value of the TyG index. For example, Cho et al. reported that the TyG index was independently associated with coronary artery disease (CAD) and obstructive CAD in non-diabetic patients. Nevertheless, no independent associations were found between the TyG index and CAD or obstructive CAD in diabetic patients (24). Furthermore, the development of acute diseases such as myocardial infarction and stroke may lead to stress-induced hyperglycemia, which can also affect the diagnostic or predictive value of the TyG index.

eGDR was initially used for assessing IR in T1DM patients, with similar accuracy to that of the HIEG clamp (13, 14). The three variables involved in calculating the eGDR include WC, HTN status, and HbA1c, which are also risk factors for CVDs. Although this indicator was initially developed among diabetic patients in the West, it has also been widely explored and applied among non-diabetic and even people of different races. A cohort study of non-diabetic populations from China indicates that eGDR may be a better predictor and intervention indicator for CVD (25). Moreover, a study of a multi-ethnic atherosclerotic population showed that the level of eGDR was linearly negatively correlated with the risk of ASCVD events (26). These studies indicate that the efficacy of eGDR as an alternative indicator of insulin resistance is universal.

The eGDR is a strong predictor of CVD occurrence in non-diabetic individuals. Incorporating the eGDR into the basic risk model can significantly improve the predictive performance of CVD, and the attributable relative risk of explainable CVD is at least partially attributed to each component in the eGDR formula (25). Another study revealed that the eGDR is linearly negatively correlated with the risk of ASCVD events, with this correlation being more pronounced in younger individuals and those without HTN (26). A study on prediabetic patients in the United States also indicated that a lower eGDR is associated with an increased incidence of CVDs and all-cause mortality (27). In this study, we also found that the eGDR is negatively correlated with the risk of CAS progression. Compared with individuals in the highest eGDR quartile group, those in the lowest quartile group had a significantly increased risk of CAS progression. These studies fully demonstrate the predictive role of the eGDR in CVDs. However, the occurrence and development of CVDs are influenced by various metabolic disorders, with lipid metabolism disorders playing crucial roles. Moreover, lipid metabolism abnormalities and glucose metabolism abnormalities often coexist (15). However, the eGDR formula does not include lipids, so the use of the eGDR alone to predict CAS progression may not be sufficient. Therefore, we used the eGDR in conjunction with the NHHR for the analysis. Non-HDL-C, LDL-C/HDL-C, TC/HDL-C, non-HDL-C/HDL-C, TG/HDL-C, and other non-traditional lipid indicators are associated with IR status and the risk of developing T2DM (28, 29). The NHHR, which represents the ratio between pro-atherosclerotic and anti-atherosclerotic components, is a good CV risk prediction indicator. Studies have shown that the NHHR is significantly associated with HTN status, CAS risk, and carotid plaque stability (22, 30, 31). In this study, the NHHR was linearly related to CAS progression and significantly mediated the effect of the eGDR on CAS progression. Additionally, further analysis revealed that the eGDR and NHHR have additive effects on CAS progression. Combining them aids in identifying high-risk individuals. However, previous studies on the eGDR have often been limited to specific populations with type 1 or type 2 diabetes. A 10-year follow-up study of 774 patients with type 1 diabetes revealed that for every 1.0-SD increase in eGDR, the risk of major CV events decreased by 44% (HR: 0.56, 95% CI: 0.39–0.80), and the risk of CAD decreased by 37% (HR: 0.63, 95% CI: 0.42–0.96) (32). Similarly, a lower eGDR is associated with an increased risk of all-cause and CVD mortality in adults with prediabetes in the United States (27). Recent studies are no longer limited to diabetic populations; regardless of diabetes status, the eGDR is associated with an increased risk of CVD and shows greater sensitivity in predicting CVD in non-diabetic individuals (25, 33). This result is consistent with the sensitivity analysis we conducted in non-diabetic patients. Additionally, the predefined groupings can affect the predictive power of the eGDR and NHHR. Subgroup analysis revealed that in the population aged >60 years, the eGDR and NHHR were not associated with the risk of CAS progression. Age-related physiological changes may play a key role in this. Age is closely related to IR risk. The reduction of muscle mass is closely related to insulin resistance and metabolic syndrome (34). Sarcopenia is widespread among the elderly population, and the incidence rate increases significantly with age (35, 36). Furthermore, a statistical study in the United States indicated that 30% of the population over 60 years old has type 2 diabetes (37). Elevated levels of inflammation in the elderly population are also widespread, even among healthy individuals. Studies have found that the circulating levels of interleukin-6, tumor necrosis factor -α and other pro-inflammatory markers increase with age (38). Atherosclerosis is also an inflammatory disease. Therefore, in elderly individuals, the increased levels of inflammatory factors with age may weaken the risks reflected by insulin resistance and lipoproteins. This result emphasizes that future research needs to pay special attention to the elderly subgroup and explore the value of incorporating sarcopenia, inflammatory indicators, etc. into the cardiovascular risk assessment model for the elderly.

This result suggests that the eGDR and NHHR, as risk factors, can be used early for specific populations, thereby having significant implications for reducing the burden of disease. The specific pathological mechanisms by which IR leads to atherosclerosis are not yet fully understood, but some studies have provided possible explanations. Impaired insulin signaling affects both the dilation and contraction functions of the vascular endothelium. IR not only leads to a deficiency of nitric oxide but also induces an increase in the synthesis of the potent vasoconstrictor endothelin-1 and reduces the availability of the vasodilator prostacyclin (39, 40). IR affects the density of LDL particles, making them more prone to oxidation and entry into the arterial intima, and increases the levels of TG-rich lipoproteins by reducing the function of lipoprotein lipase, further leading to the formation of atherosclerotic plaques (40, 41). Moreover, IR can activate the NF-κB pathway to trigger an inflammatory response, ultimately leading to CV events (42, 43). This study aims to clarify the predictive value of eGDR and the NHHR for the progression of carotid atherosclerosis. The results of this study are helpful for physicians to make clinical surgical decisions, that is, by early identification of high-risk patients (such as those with rapid plaque progression), to optimize the intervention timing and postoperative management of carotid revascularization.

### Strengths and limitations

This study has several advantages. This was a large-scale longitudinal cohort study in a real-world setting. Repeated carotid ultrasound and biochemical index measurements allowed us to explore the impact of the eGDR and NHHR on the progression of CAS in adults. Our research has several limitations that need to be noted. First, this study was conducted at the Chinese PLA General Hospital, and the included research population was mainly the physical examination population, most of whom were of Han ethnicity. Therefore, the external validity of the results of this study may be limited, and caution is needed when applying them to other geographical regions or populations with different demographic characteristics. To confirm the general applicability of the findings of this study and enhance their external validity, future research should focus on validating these results in prospective cohorts of multi-center and more diverse populations. Second, as an observational study, we cannot determine the causal relationships between variables and outcomes. Although, we conducted a mediation analysis to approximate the causal relationship. This study can only confirm the association between eGDR and CAS. In future studies, whether improving eGDR can delay carotid atherosclerosis still needs to be verified through lifestyle intervention trials. Third, although the sample size of this study was relatively large, the number of elderly individuals was relatively small, which may affect the generalizability of our conclusions. Therefore, it is necessary to conduct statistical analysis on a broader population sample to further confirm our results. In addition, our eGDR and NHHR metrics are calculated from baseline levels.

## Conclusions

This study revealed that simultaneously assessing the eGDR and NHHR can more comprehensively reflect long-term CAS risk in the population (especially among young and middle-aged individuals). Clinicians should comprehensively monitor the dynamic changes in the eGDR and NHHR during routine health check-ups to improve the identification of high-risk populations and develop more effective treatment measures.

## Data Availability

The data analyzed in this study is subject to the following licenses/restrictions: The datasets used or analysed during the current study are available from the corresponding author on reasonable request. Requests to access these datasets should be directed to Qiang Zeng, zq301@126.com.
