# Correction: Estimated glucose disposal rate and non-HDL-c/HDL-c ratio with the progression of carotid atherosclerosis: a long-term cohort study

**DOI:** 10.3389/fmed.2025.1704381

**Published:** 2025-10-02

**Authors:** Bingqing Han, Jing Ma, Shanshan Liu, Chao Fu, Hao Zhang, Yi Luo, Fei Wang, Qiang Zeng

**Affiliations:** ^1^School of Medicine, Nankai University, Tianjin, China; ^2^Health Management Institute, The Second Medical Center & National Clinical Research Center for Geriatric Diseases, Chinese PLA General Hospital, Beijing, China

**Keywords:** estimated glucose disposal rate, Non-HDL-c/HDL-c ratio, insulin resistance, carotid atherosclerosis, metabolism

Affiliation From the School of Medicine, Nankai University, Tianjin, China was omitted for the following author Qiang Zeng.

The original version of this article has been updated.

