# Supplementary material for: Estimated glucose disposal rate and non-HDL-c/HDL-c ratio with the progression of carotid atherosclerosis: a long-term cohort study
Source: Front Med (Lausanne). 2025 Jul 17;12:1627246. doi: 10.3389/fmed.2025.1627246 (PMC12310652; doi:10.3389/fmed.2025.1627246)
Supplement: Supplementary file 1 [file Data_Sheet_1.docx]

Supplementary material

**Table S1.** Baseline characteristics of participants by outcomes

| **Characteristics** | **Without CAS progression** | **CAS progression** | **Overall** | ***P* value** |
| --- | --- | --- | --- | --- |
|  | **(N=3609)** | **(N=3751)** | **(N=7360)** |  |
| Femal,n(%) | 1439 (39.90%) | 844 (22.50%) | 2283 (31.00%) | <0.001 |
| Age, years | 47.9 ± 9.26 | 50.7 ± 7.92 | 49.3 ± 8.72 | <0.001 |
| Hypertention,n(%) | 1296 (35.91%) | 1961 (52.28%) | 3257 (44.25%) | <0.001 |
| Diabetes,n(%) | 550 (15.24%) | 920 (24.53%) | 1470 (19.97%) | <0.001 |
| SBP, mmHg | 119 ± 18.70 | 124 ± 17.70 | 121 ± 18.40 | <0.001 |
| DBP, mmHg | 78.2 ± 11.70 | 80.3 ± 11.50 | 79.3 ± 11.70 | <0.001 |
| Current Smoking,n(%) | 1142 (31.64%) | 1599 (42.63%) | 2741 (37.24%) | <0.001 |
| Current Drinking,n(%) | 2015 (55.83%) | 2486 (66.28%) | 4501 (61.15%) | <0.001 |
| BMI, kg/m2 | 24.9 ± 3.71 | 26.2 ± 3.45 | 25.6 ± 3.65 | <0.001 |
| WC, cm | 87.0 ± 12.50 | 91.9 ± 10.70 | 89.5 ± 11.90 | <0.001 |
| eGDR | 8.96 ± 2.46 | 7.84 ± 2.28 | 8.39 ± 2.43 | <0.001 |
| NHHR | 2.65 (0.472−7.21) | 3.16 (0.40−7.96) | 2.89 (0.40−7.96) | <0.001 |
| FBG, mmol/L | 5.55 ± 1.26 | 5.90 ± 1.54 | 5.73 ± 1.42 | <0.001 |
| HbA1c,% | 5.71 ± 0.77 | 5.93 ± 0.93 | 5.82 ± 0.86 | <0.001 |
| Hemoglobin,g/L | 143 ± 16.40 | 148 ± 14.40 | 145 ± 15.50 | <0.001 |
| TC, mmol/L | 4.62 ± 0.90 | 4.76 ± 0.95 | 4.69 ± 0.93 | <0.001 |
| TG, mmol/L | 1.30 (0.28−9.21) | 1.58 (0.38−15.50) | 1.45 (0.28−15.50) | <0.001 |
| LDL-C, mmol/L | 3.03 ± 0.82 | 3.16 ± 0.87 | 3.10 ± 0.85 | <0.001 |
| HDL-C, mmol/L | 1.30 ± 0.37 | 1.18 ± 0.33 | 1.24 ± 0.35 | <0.001 |
| UA,μmol/L | 335 ± 91.80 | 359 ± 85.90 | 347 ± 89.60 | <0.001 |
| hsCRP,mg/L | 0.10 (0−17.70) | 0.12 (0−16.50) | 0.11 (0−17.70) | <0.001 |
| Lipid-lowering Medications,n(%) | 219(6.0%) | 272(7.8%) | 489(6.6%) | 0.126 |
| Antidiabetic Medications,n(%) | 293(8.1%) | 496(13.2%) | 789(10.7%) | <0.001 |

BMI, body mass index; DBP, diastolic blood pressure; SBP, systolic blood pressure; eGDR, estimated glucose disposal rate; NHHR, non-HDL-C/HDL-C ratio; FBG, fasting blood glucose; HbA1c, glycosylated hemoglobin A1c; HDL-C, high density lipoprotein cholesterol; hsCRP, high-sensitivity C-reactive protein; LDL-C, low density lipoprotein cholesterol;TC, total cholesterol; TG, triglycerides; UA, uric acid; WC, waist circumference

**Table S2**. The association of eGDR (defined hypertension based on 130/80 mmHg) and NHHR with CAS progression among participants.

|  | Unadjusted | | Model 1 | | Model 2 | |
| --- | --- | --- | --- | --- | --- | --- |
|  | HR (95% CI) | *P* Value | HR (95% CI) | *P* Value | HR (95% CI) | *P* Value |
| eGDR ≥ median & NHHR < 2.89 | Reference |  | Reference |  | Reference |  |
| eGDR ≥ median & NHHR ≥ 2.89 | 1.37(1.24-1.50) | ＜0.001 | 1.18(1.07-1.30) | 0.001 | 1.10(0.99-1.22) | 0.079 |
| eGDR < median & NHHR < 2.89 | 1.56(1.42-1.73) | ＜0.001 | 1.25(1.13-1.39) | ＜0.001 | 1.18(1.06-1.31) | 0.003 |
| eGDR < median & NHHR ≥ 2.89 | 1.76(1.61-1.92) | ＜0.001 | 1.42(1.30-1.56) | ＜0.001 | 1.27(1.15-1.41) | ＜0.001 |

Model 1: Adjusted for age and male; Model 2: Adjusted for age, sex, BMI, current smoking, current drinking, TG, HGB, UA, hs-CRP. CAS, carotid atherosclerosis progression; eGDR, estimated glucose disposal rate; NHHR, non-HDL-C/HDL-C ratio. HGB, Hemoglobin; hsCRP, high-sensitivity C-reactive protein; HR, hazard ratio; UA, uric acid; CI, confdence interval; median of eGDR: 8.711.

**Table S3**. The association of eGDR and NHHR（Participants who did not take hypoglycemic drugs or lipid-lowering drugs）with CAS progression among participants.

|  | Unadjusted | | Model 1 | | Model 2 | |
| --- | --- | --- | --- | --- | --- | --- |
|  | HR (95% CI) | *P* Value | HR (95% CI) | *P* Value | HR (95% CI) | *P* Value |
| eGDR ≥ median & NHHR < 2.89 | Reference |  | Reference |  | Reference |  |
| eGDR ≥ median & NHHR ≥ 2.89 | 1.46(1.30-1.63) | ＜0.001 | 1.25(1.12-1.40) | 0.001 | 1.17(1.04-1.32) | 0.011 |
| eGDR < median & NHHR < 2.89 | 1.70(1.53-1.90) | ＜0.001 | 1.30(1.16-1.46) | ＜0.001 | 1.21(1.07-1.37) | 0.003 |
| eGDR < median & NHHR ≥ 2.89 | 1.88(1.71-2.06) | ＜0.001 | 1.43(1.29-1.59) | ＜0.001 | 1.26(1.12-1.42) | ＜0.001 |

Model 1: Adjusted for age and male; Model 2: Adjusted for age, sex, BMI, current smoking, current drinking, TG, HGB, UA, hs-CRP. CAS, carotid atherosclerosis progression; eGDR, estimated glucose disposal rate; NHHR, non-HDL-C/HDL-C ratio. HGB, Hemoglobin; hsCRP, high-sensitivity C-reactive protein; HR, hazard ratio; UA, uric acid; CI, confdence interval; median of eGDR: 8.711.

**Table S4**. The association of eGDR and NHHR（Participants with a follow-up period of less than 12 months or more than 96 months were excluded）with CAS progression among participants.

|  | Unadjusted | | Model 1 | | Model 2 | |
| --- | --- | --- | --- | --- | --- | --- |
|  | HR (95% CI) | *P* Value | HR (95% CI) | *P* Value | HR (95% CI) | *P* Value |
| eGDR ≥ median & NHHR < 2.89 | Reference |  | Reference |  | Reference |  |
| eGDR ≥ median & NHHR ≥ 2.89 | 1.46(1.30-1.64) | ＜0.001 | 1.28(1.13-1.44) | 0.001 | 1.17(1.03-1.33) | 0.011 |
| eGDR < median & NHHR < 2.89 | 1.75(1.57-1.95) | ＜0.001 | 1.37(1.22-1.53) | ＜0.001 | 1.28(1.13-1.44) | 0.003 |
| eGDR < median & NHHR ≥ 2.89 | 1.91(1.73-2.11) | ＜0.001 | 1.50(1.35-1.67) | ＜0.001 | 1.31(1.66-1.48) | ＜0.001 |

Model 1: Adjusted for age and male; Model 2: Adjusted for age, sex, BMI, current smoking, current drinking, TG, HGB, UA, hs-CRP. CAS, carotid atherosclerosis progression; eGDR, estimated glucose disposal rate; NHHR, non-HDL-C/HDL-C ratio. HGB, Hemoglobin; hsCRP, high-sensitivity C-reactive protein; HR, hazard ratio; UA, uric acid; CI, confdence interval; median of eGDR: 8.711.


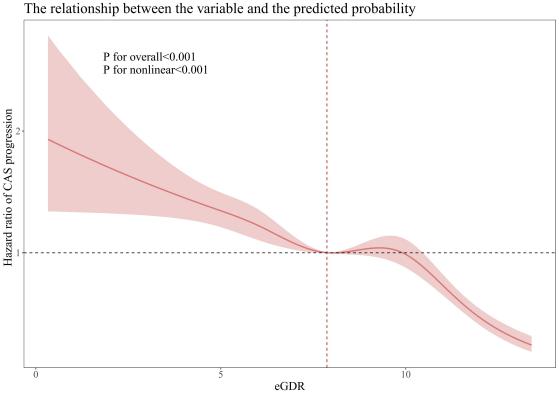

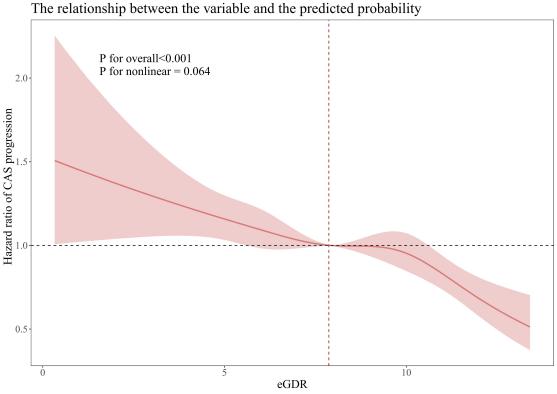


Fig. S1 Restricted cubic spline curves for CAS progression according to the eGDR and NHHR. Hazard ratios are indicated by solid lines and 95% CIs by shaded areas. The horizontal dotted line represents the hazard ratio of 1.0. The adjusted models age, sex, BMI, current smoking, current drinking, TG, HGB, UA, hs-CRP. eGDR, estimated glucose disposal rate; NHHR, non-HDL-C/HDL-C ratio;


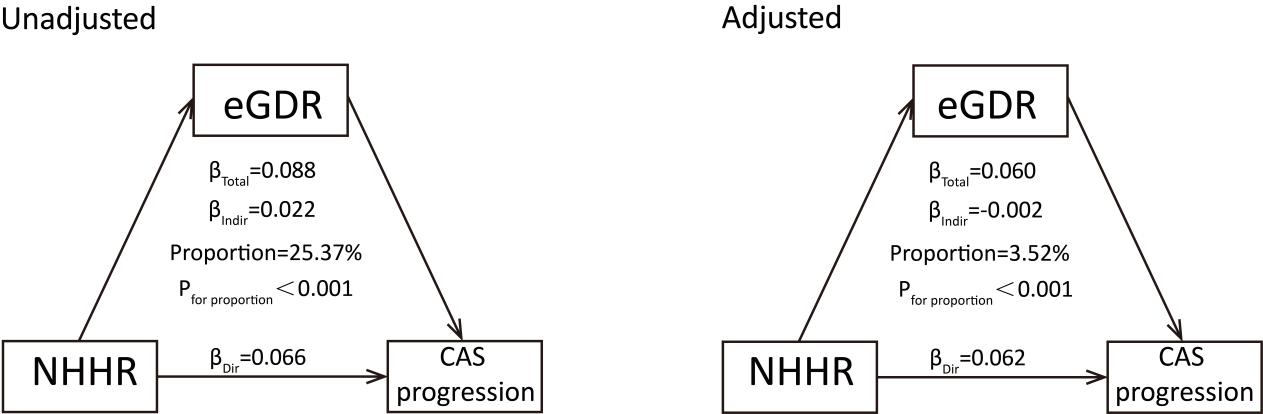


Fig. S2 Mutual mediation efects of the eGDR and NHHR on CAS progression. Adjusted for age, sex, BMI, current smoking, current drinking, TG, HGB, UA, hs-CRP. CAS, carotid atherosclerosis progression; eGDR, estimated glucose disposal rate; HGB, Hemoglobin; hsCRP, high-sensitivity C-reactive protein; NHHR, non-HDL-C/HDL-C ratio;

**
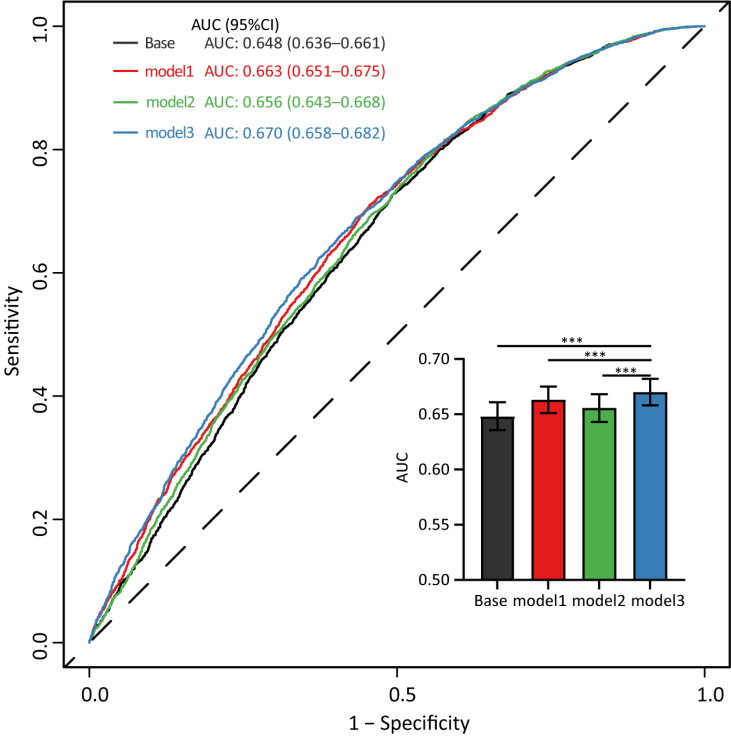
**

Figure S3. Receiver operating characteristic curves of the eGDR, NHHR, and eGDR+NHHR for CAS. The basic model adjusted age, sex, current smoking, current drinking, TC, HDL, TG, LDL, UA, hsCRP; model 1: basic model+eGDR; model 2: basic model+NHHR; model 2: basic model+eGDR+NHHR; BMI, body mass index; DBP, diastolic blood pressure; SBP, systolic blood pressure; eGDR, estimated glucose disposal rate; NHHR, non-HDL-C/HDL-C ratio; FBG, fasting blood glucose; HbA1c, glycosylated hemoglobin A1c; HDL-C, high density lipoprotein cholesterol; hsCRP, high-sensitivity C-reactive protein; LDL-C, low density lipoprotein cholesterol;TC, total cholesterol; TG, triglycerides; UA, uric acid; WC, waist circumference
